# Aberrant Salience Across Levels of Processing in Positive and Negative Schizotypy

**DOI:** 10.3389/fpsyg.2019.02073

**Published:** 2019-09-18

**Authors:** Charlotte A. Chun, Peter Brugger, Thomas R. Kwapil

**Affiliations:** ^1^Department of Psychology, University of North Carolina at Greensboro, Greensboro, NC, United States; ^2^Department of Psychology, Temple University, Philadelphia, PA, United States; ^3^Neuropsychology Unit, Department of Neurology, University Hospital Zürich, Zurich, Switzerland; ^4^Department of Psychology, University of Illinois at Urbana-Champaign, Champaign, IL, United States

**Keywords:** aberrant salience, schizotypy, latent inhibition, contingency illusions, superstitious behavior

## Abstract

Schizotypy is a multidimensional construct conceptualized as the expression of the underlying vulnerability for schizophrenia. Certain traits of positive schizotypy, such as odd beliefs, unusual perceptual experiences, suspiciousness, and referential thinking show associations with aberrant salience. Positive schizotypy may involve hyper-attribution of salience toward insignificant events, whereas negative schizotypy may involve hypo-attribution of salience, even toward important events. Attribution of salience is thought to involve dopamine-mediated processes, a mechanism that is disrupted in schizotypy; however, little is known about the cognitive processes potentially underlying salience attribution. The present study assessed the relationship between aberrant salience and latent inhibition (LI), as well as their associations with positive and negative schizotypy. Salience was measured at various stages of processing, including visual salience, attributions of salience to contingency illusions, and self-reported experience of salience. Schizotypy traits were differentially associated with self-reported aberrant salience experiences: positive schizotypy showed positive associations (β = 0.67, *f^2^* = 0.82, large effect) and negative schizotypy showed inverse associations (β = −0.20, *f^2^* = 0.07, small effect). However, neither schizotypy dimension was associated with visual salience, contingency illusions, or LI. Salience processing across perceptual, cognitive, and experiential levels likely involves different mechanisms, some of which may not show major disruption in subclinical manifestations of schizotypy.

## Introduction

The current study assessed the association between schizotypy and aberrant salience. We aimed to measure salience using a variety of methods, including perceptual and behavioral tasks, and self-report measures. In this section, we provide a brief overview of salience theories from a variety of perspectives including clinical, phenomenological, neural, cognitive, and behavioral science. An in-depth account of each perspective is beyond the scope of this paper; rather, our aim was to set the stage for the interdisciplinary assessment approach used in this study.

### Schizotypy

Schizotypy refers to the expression of the developmental vulnerability for schizophrenia that ranges along a continuum from subclinical traits to clinical disorders (Meehl, 1990; Claridge, 1997; Lenzenweger, 2010; Kwapil and Barrantes-Vidal, 2012). Schizophrenia and schizotypy are heterogeneous, and multidimensional models commonly include positive, negative, and disorganized dimensions (e.g., Fonseca-Pedrero et al., 2018; Kwapil et al., 2018). The current study focused specifically on positive and negative schizotypy. Positive schizotypy is characterized by odd beliefs and unusual perceptual experiences that range from mild, brief experiences that minimally impact functioning to delusions and hallucinations seen in psychotic disorders. In its milder forms, it may even encompass personality traits better designated “adaptive” than psychosis-prone (e.g., Leonhard and Brugger, 1998; Holt, 2015). Negative schizotypy is characterized by diminished functioning, such as flattened affect, anhedonia, loss of volition, cognitive deficits, and decreased social interest that range from minimal impairment to debilitating symptoms seen in schizophrenia (Millan et al., 2014). Positive and negative schizotypy are associated with differential patterns of symptoms and impairment across a wide array of domains (Kwapil and Barrantes-Vidal, 2015).

### Aberrant Salience

Aberrant salience provides a theoretical framework for understanding the development, onset, and presentation of, specifically, positive symptoms of schizotypy and schizophrenia (Kapur, 2003; Kapur et al., 2005). Although various operationalizations have been offered, salience generally refers to the distinctiveness of a stimulus, due either to its physical properties or to affective and motivational factors (e.g., Itti and Koch, 2000; Kapur, 2003). Kapur described aberrant salience as the process of placing inappropriate significance on neutral events. For example, a person with subclinical schizotypy might wonder whether it is a sign that someone left a newspaper open to a story about email hacking. A person experiencing active psychotic symptoms may see the same occurrence and be convinced it is a warning that someone is monitoring their electronic communication. Thus, the severity of aberrant salience may, in part, drive the severity of psychotic and psychotic-like symptoms. Kapur’s aberrant salience model suggests that the onset of positive symptoms can occur gradually across a pre-psychotic period of heightened awareness, characterized by increasing assignment of marked and deviant importance to internal and external stimuli. In anticipation of this model, early phenomenologists described the key features of this pre-psychotic stage as “apophenia,” i.e., the “unmotivated seeing of connections” which would be accompanied by the “specific experience of an abnormal meaningfulness” (Conrad, 1958, p. 46).

### Aberrant Salience Across Levels of Processing

The term salience has been used to describe a range of phenomena across various levels of processing; however, it is unclear whether these phenomena labeled as “salience-related constructs” actually involve the same processes. The controversy surrounding abnormal significance and the level of awareness at which it manifests dates back to the early phenomenological literature, before the term aberrant salience was ever used. Gruhle (1915) described abnormal significance as occurring at the level of conscious thought rather than perception. However, theorists such as Conrad (1958) and Matussek (1952) rejected this idea (see also Mishara, 2010). According to Matussek (1952), pg. 98), “Abnormal significance is perceptually encountered as an integral part of the object. It is not primarily deduced, thought out, or dredged up in some other way from our thoughts, but rather experienced directly as inherent in the object.” Kapur, whose theory aligns more closely with Gruhle’s, stated that his operationalization of salience occurs “at a level of cognitive associations, reward and reinforcement, and motivational significance” (Kapur, 2003, pg. 18).

Some neurocognitive psychologists theorize that automatic processing may reasonably account for altered perception (e.g., Gray et al., 1991; Fletcher and Frith, 2009) and afterward serve to burden controlled processing (e.g., Nuechterlein and Dawson, 1984). That is, perception and belief may not be entirely separable, and automatic processes such as errors in predictive coding may contribute to alterations in both perception and belief. Hemsley and Gray proposed a cognitive model for schizophrenia in which a deficit in integrating sensory input with the context of stored memories leads to prediction errors and subsequent attentional focus on aspects of the environment that are usually ignored (e.g., Hemsley, 1987, 1993; Gray et al., 1991; see Hemsley, 2005 for review of the Gray-Hemsley model and related theories). Fletcher and Frith further described how experiences of external control may arise as disconnectivity among sensory regions in the brain leads to difficulty differentiating among internal and external stimuli, resulting in faulty prediction of events. Put simply, these prediction errors signal learning and, in conjunction with impairments in probabilistic reasoning, may contribute to interpretations that internally generated thoughts or movements are coming from outside oneself (Fletcher and Frith, 2009). Overall, theorists disagree on the relative roles that perceptual and inferential abnormalities play in contributing to experiences of aberrant salience and delusional beliefs but there is some evidence that these processes may be intertwined. However, pre-attentive and attentive processes are not necessarily conceptualized in the framework of predictive coding.

#### Pre-attentive and Attentive Processes

Normal attribution of salience in healthy individuals facilitates efficient processing and evaluation of stimuli. Cognitive psychologists and neuropsychologists view visual salience as a feature involving pre-attentional processing; a stimulus is salient if it is visually distinct from its surroundings, thereby eliciting selective attention (e.g., Koch and Ullman, 1985). This selection occurs when separate, pre-attentive features (e.g., color, luminance, and spatial orientation) that make a stimulus distinctive are simultaneously processed in the visual cortex and combined onto a saliency map (Koch and Ullman, 1985; Itti and Koch, 2000, 2001; Itti, 2007). Though not as well studied, a comparable process may occur in auditory perception (Kayser et al., 2005). Pre-attentive features are integrated on the saliency map to form a distinctive unit of perceptual salience that can be modulated by attentional and emotional processing. Top-down modulation occurs when information from higher-order processing influences the importance of certain features, such as color, so that if you are searching for a blue shirt in a pile of clothing, for example, blue stimuli will be more salient (Wolfe et al., 1989; Wolfe, 1994; Itti et al., 1998). This higher-order salience is often termed incentive or motivational salience – the interaction of perception and motivation that makes a stimulus focus our attention because it represents something we want. In sum, a stimulus can be attentionally selected because its low-level features are distinctive or because it is relevant to a higher-level criterion (e.g., personal expectations or a task at hand).

Positive, but not negative, schizotypy is associated with over-attribution of salience to stimuli in laboratory tasks. For example, positive schizotypy was associated with hearing illusory speech during white noise tasks and increased skin conductance response to unconditioned stimuli, suggesting aberrant response to neutral events. People with negative schizotypy did not report speech illusions and showed decreased autonomic response to salient stimuli (Galdos et al., 2010; Balog et al., 2013). These findings highlight the importance of examining associations separately for positive and negative schizotypy – treating schizotypy as a homogenous construct may have masked these contrasting associations.

As noted above, visual salience appears to involve pre-attentive and attentive processes; however, there have been mixed results for tasks assessing attentional capture by visually salient stimuli. One study showed that gamma oscillation increases in response to salient distractors were correlated with schizotypy scores; however, behavioral responses were unassociated (Kornmayer et al., 2015). Tsakanikos (2004) failed to find evidence of increased sensitivity to salience in schizotypy using a visual pop out task. However, this study used small samples with an extreme groups design and did not differentiate positive and negative schizotypy. Thus, the current study sought to examine salience using a visual pop out task in relation to a multidimensional model of schizotypy.

The experience of aberrant salience has been described in terms of difficulty screening out irrelevant details. As one patient recounted, “I had very little ability to sort the relevant from the irrelevant. The filter had broken down. Completely unrelated events became intricately connected in my mind” (MacDonald, 1960, p. 219). This filtering of details can be approximated using latent inhibition (LI) tasks. LI is a pre-attentional phenomenon found in the general population in which familiar stimuli take longer to acquire new meaning than new stimuli. Previous research showed reduced LI in schizophrenia-spectrum psychopathology (e.g., Tsakanikos, 2004; Lubow, 2005). Individuals with positive—but not negative—schizotypy show reduced LI, indicative of impaired ability to screen out pre-exposed stimuli for use in a new task (e.g., Evans et al., 2007; Kumari and Ettinger, 2010; Granger et al., 2012).

#### Neural Processes

The dopamine hypothesis indicates that abnormal dopamine transmission is differentially implicated in schizophrenia symptoms. Broadly, diminished dopaminergic transmission, especially in prefrontal areas, is associated with negative symptoms, whereas hyperdopaminergic functioning, especially in striatal and limbic areas, is associated with positive symptoms (e.g., Abi-Dargham, 2012). Dopamine modulates intrinsic functional connectivity between striatal and cortical regions, and neuroimaging studies indicate that these connections are disrupted in patients with schizophrenia (Horga et al., 2016). Early abnormalities may also be present in subclinical schizotypy: one study found that hyperdopaminergic states (induced by L-DOPA) and positive schizotypy traits (independent of L-DOPA) showed functional decoupling between striatal and occipitotemporal regions during resting state. In contrast, dopamine may have helpful effects on negative schizotypy: negative traits were associated with coupling in the group that received L-DOPA, compared to decoupling in the placebo group (Rössler et al., 2018). These findings support the hypothesis that disconnectivity of sensory information from the striatum may be implicated in the dopamine-driven salience attribution processes that go awry in schizophrenia-spectrum psychopathology (Winton-Brown et al., 2014; Rössler et al., 2018).

Dopamine systems mediate motivational salience by transforming a neutral stimulus into a neural representation of reward. These systems can then interact with associative learning through classical conditioning, so that a conditioned stimulus gains motivational salience as it becomes associated with reward or punishment (Berridge and Robinson, 1998). Reduced dopamine in the mesolimbic pathway is associated with a diminished ability to mediate motivational salience in reward processing and is proposed to contribute to motivational deficits in patients with negative schizophrenia (Juckel et al., 2006; Heinz and Schlagenhauf, 2010). Researchers employing classical conditioning paradigms to test disrupted motivational salience in schizophrenia have shown alterations in neural and autonomic response associated with reward learning in patients with schizophrenia (Jensen et al., 2008; Walter et al., 2009). Disrupted reward learning may contribute to both increased aberrant salience associated with positive symptoms and diminished aberrant salience associated with negative symptoms.

The neural salience network, which is involved in identifying relevant information, is primarily composed of the anterior insula and anterior cingulate. Neuroanatomical evidence suggests that patients with schizophrenia have reduced gray matter in these regions and that these structural deficits are associated with symptoms of reality distortion (Palaniyappan et al., 2010). Functional studies have shown reduced functional connectivity between the proposed salience network and reward processing system in patients with negative symptoms (Gradin et al., 2013). In sum, schizophrenia-spectrum psychopathology is associated with alterations in structure, function, and neurotransmission of the neural networks and processes involved in salience attribution.

#### Cognitive Associations and Causality

Superstitious beliefs are “false conceptions of causation” (Brugger and Viaud-Delmon, 2010, p. 252), which occur regularly in the general population and are often culturally relevant. Superstitious beliefs exist along a continuum and variations in superstitiousness may reflect differences in the amount of evidence individuals require to accept hypotheses (Brugger and Graves, 1997). Superstitious beliefs are distinct from superstitious behaviors, as demonstrated in previous research using a task that assesses participants’ likelihood of perceiving causal relationships in response to reward learning. In this task, superstitious behaviors are demonstrated by idiosyncratic behavior that is irrelevant to the true cause of reward, whereas superstitious beliefs are demonstrated by believing in illusory contingencies without hypothesis testing (Brugger and Graves, 1997; Brugger and Viaud-Delmon, 2010). On an illusory contingency task, people high in magical ideation engaged in comparable superstitious behaviors to those low in magical ideation; however, they had more superstitious beliefs. Namely, they tested fewer hypotheses and held greater beliefs about causal relationships that they had never tested (Brugger and Graves, 1997). Even when the number of superstitious actions are experimentally controlled, people high in self-reported superstitious beliefs are more likely to perceive non-contingent events as causal (Griffiths et al., 2018). With this in mind, the current study specifically examined interpretations of causality in relation to aberrant salience.

The experience of apophenia, or perceiving patterns or connections in random events, has been demonstrated in empirical studies. One study showed that a neurophysiological measure of automatic attention to early auditory processing was associated with a tendency toward finding meaningful patterns in random stimuli. Additionally, positive schizotypy was associated with the self-reported tendency to experience coincidences in one’s life as meaningful (Rominger et al., 2018). Another study found that positive schizotypy was associated with false positives on a semantic association task, such that participants high in positive traits were more likely to label unrelated item pairs as related (Blain et al., 2019). Experience sampling studies have captured subjective reports of altered experience of salience in real-world settings. For example, one study showed that positive schizotypy is associated with referential ideas in daily life, such as the feeling that familiar things have special meaning, whereas negative schizotypy is associated with diminished thoughts or emotions in daily life (Barrantes-Vidal et al., 2013).

### Assessment of Aberrant Salience

Cicero et al. (2010) developed the aberrant salience inventory (ASI), a self-report measure designed to assess aspects of aberrant salience in line with Kapur’s theory. The ASI was significantly correlated with positive schizotypy traits of magical ideation, perceptual aberration, referential thinking, dissociation, and absorption, but was not associated with the negative schizotypy trait of social anhedonia (Cicero et al., 2010). However, associations with positive schizotypy measures may be confounded in part by the similarity in content, especially regarding referential beliefs, between the ASI and positive schizotypy scales.

### Summary and Rationale for the Current Study

The aberrant salience hypothesis provides a framework for understanding the development, onset, and manifestation of positive symptoms. Disruptions in salience attribution are found in subclinical and clinical schizotypy across different levels of processing using a variety of laboratory and real-world measures. However, the term “salience” is used so widely in the literature to describe phenomena across very different levels of processing (e.g., neural, cognitive, behavioral) that it becomes unclear to what extent these constructs overlap. Further, the mechanisms underlying faulty attributions of significance or causality are not fully understood. If we are to extend our theoretical understanding of the development of psychotic symptoms, it is important to describe the connections among salience constructs and examine underlying processes that may be involved in attributions of salience.

The current study examined the associations of: (a) salience measures across different levels of cognitive processing, (b) salience measures with LI, (c) continuous measures of positive and negative schizotypy with salience measures on questionnaire and laboratory tasks, and (d) positive and negative schizotypy with LI. It was hypothesized that salience would not present as a unitary process across different levels of processing. That is, beliefs about contingency and self-report of aberrant salience, which involve greater levels of reasoning and belief, were expected to correlate more strongly with one another than with visual salience.

It was expected that positive and negative schizotypy would be differentially associated with deviant attributions of salience. Specifically, it was hypothesized that positive schizotypy would be associated with higher ASI scores, greater contingency illusions, and greater sensitivity to visual salience. It was expected that negative schizotypy would be associated with lower ASI scores and diminished sensitivity to visual salience but would be unassociated with contingency illusions. Given the hypothesized pattern of differential associations, we also expected to find significant positive x negative schizotypy interactions in the prediction of the salience measures (over-and-above the main effects of positive and negative schizotypy). Specifically, we expected that negative schizotypy would moderate the association of positive schizotypy with the salience and illusory contingency measures, such that stronger effects for positive schizotypy would be found at lower levels of negative schizotypy (especially for ASI scores and with belief in more spurious rules and consideration of more rules that were never tested on the illusory contingency task). It was hypothesized that LI would correlate negatively with salience measures but it was expected to show stronger associations with visual salience than with contingency illusions and self-reported aberrant salience. It was expected that positive schizotypy would be inversely associated with LI and that negative schizotypy would be unassociated.

## Materials and Methods

### Participants and Procedures

Participants were recruited from a pool of college undergraduates enrolled in psychology courses. Students enrolled in these classes had the option to sign up to participate in experiments to receive course credit. The study received IRB approval and participants provided informed consent. Questionnaires and laboratory tasks were administered electronically in group format. Individual items within schizotypy and salience scales were administered in fixed order but the order of the measures was randomized. Data was collected on 100 participants and 16 were excluded for the following reasons: 7 with low visual salience performance, 5 with low LI performance, 2 with low performance on both visual salience and LI, 1 with an elevated score on an infrequent responding scale, and 1 with missing data. Analyses were run on the final included sample of 84 participants. The final sample had a mean age of 19.4 years (SD = 2.6, range = 18–33), was 69% female, 50% White, 13% Multiethnic, 13% Asian-American/Pacific Islander, 11% Black, 6% Hispanic/Latino, and 2% Native American. Four percent of participants did not indicate their ethnicity.

### Materials

#### Schizotypy Measures

Participants completed the brief forms of the Wisconsin Schizotypy Scales (Winterstein et al., 2011). The Perceptual Aberration Scale assesses odd perceptual experiences and bodily distortions (Chapman et al., 1978), the Magical Ideation Scale assesses magical thinking and belief in improbable ideas (Eckblad and Chapman, 1983), the Physical Anhedonia Scale assesses deficits in sensory and aesthetic pleasure (Chapman et al., 1976), and the Revised Social Anhedonia Scale assesses asociality and diminished pleasure in social interactions (Eckblad et al., 1982). These scales reliably produce two factors, positive and negative schizotypy, that account for 80% of their variance. Positive and negative schizotypy factor scores were computed using formulae based upon norms from 6,137 young adults (Gross et al., 2015). The schizotypy dimensions from the short forms have good reliability, correlated highly with the original scales, and demonstrated validity through expected associations with other questionnaire and interview measures (Gross et al., 2012, 2015). Internal consistency values for the four short scales range from Cronbach’s α values of 0.62 to 0.83 (Winterstein et al., 2011). A 13-item infrequency scale (Chapman and Chapman, 1983) was interspersed with the schizotypy items to screen out invalid responding. One participant was excluded based on the *a priori* decision to exclude participants who endorsed 3 or more infrequency items.

#### Aberrant Salience Inventory

The ASI (Cicero et al., 2010) is a self-report questionnaire that produces a total score and five subscale scores for aberrant salience: increased feelings of significance, anomalies of perception, impending understanding, heightened emotionality, and heightened cognition. The ASI has good internal consistency (Cronbach’s α = 0.89) and shows convergent and discriminant validity with other schizotypy and personality measures (Cicero et al., 2010).

#### Illusory Contingency

**FIGURE 1:**
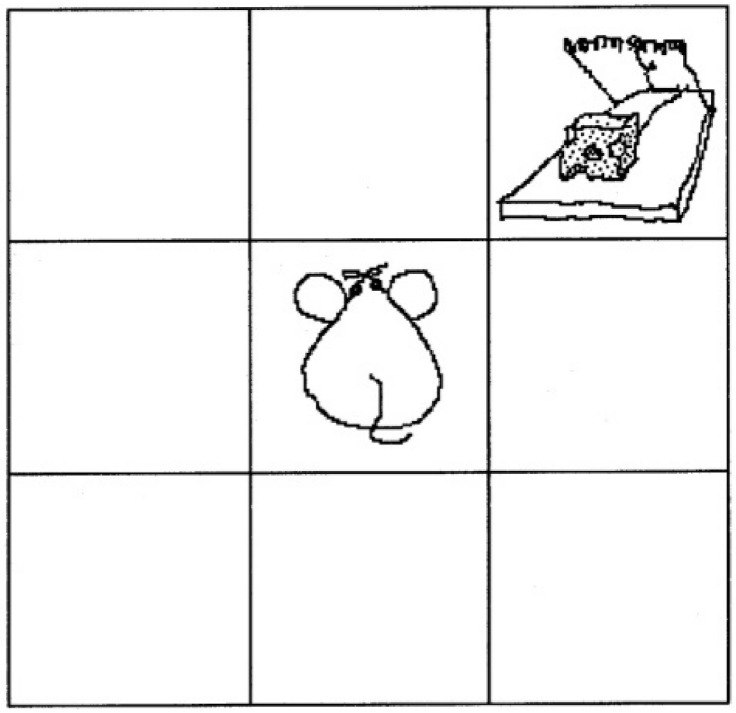
Illusory Contingency Task from Brugger and Graves (1997). In this game, participants must try to obtain the cheese by moving the mouse avatar around the matrix using the direction arrows on the keyboard. They must figure out the hidden rule that determines whether they get the cheese or get caught by the mousetrap once they reach the target.

The Illusory Contingency task (Brugger and Graves, 1997) was designed to assess superstitious behavior and beliefs. Illusions of contingency on this task are associated with scores on the Magical Ideation Scale (Brugger and Viaud-Delmon, 2010). The task was presented as a computer game in which participants try to get a piece of cheese in a mousetrap by moving a mouse avatar up, down, left, and right in a matrix using arrow keys on the computer keyboard (see Figure 1). The sole rule is that trials in which the participant reaches the cheese after 4 s yield success, followed by a screen stating, “Got it!” Trials in which the participant reaches the cheese in 4 s or less yield failure, followed by a screen stating, “Trapped!” Participants were not informed about the 4-second rule. After 100 trials, the game ended and participants completed a questionnaire about their experience. They were asked to freely list the rule(s) they believe determined whether they successfully got the cheese and to rate their certainty using a 7-point Likert scale. Next, participants were asked which of the following nine rules they considered at any point during the task and which they specifically tested and ruled out: (1) begin the sequence with a particular key press, (2) avoid jumping on one or more particular squares, (3) avoid pressing a certain arrow key, (4) step on a particular square right before jumping on the trap, (5) repeat a path a certain number of times, (6) touch a certain number of squares, (7) wait a certain amount of time before jumping on the trap (i.e., the only rule actually present), (8) step on each square exactly once, and (9) step on each square twice. The following variables were computed as measures of superstitious beliefs: number of incorrect rules listed freely (before being presented with the list of possible rules), confidence in incorrect rules that were listed freely, number of incorrect rules tested and ruled out from the list of possible rules, and number of rules from the possible list that were considered but never tested. Additional variables were computed to describe basic task outcomes and compare to previous research: number of successful trials, average time per trial, number of ineffective key presses (key presses that do not result in a movement), average path length (number of movements without ineffective key presses), and whether the correct rule was freely listed. Thus, only the interpretations of causality described on the questionnaire were used as predictors of aberrant salience, not the behavioral measures from task performance.

#### Visual Salience

**FIGURE 2:**
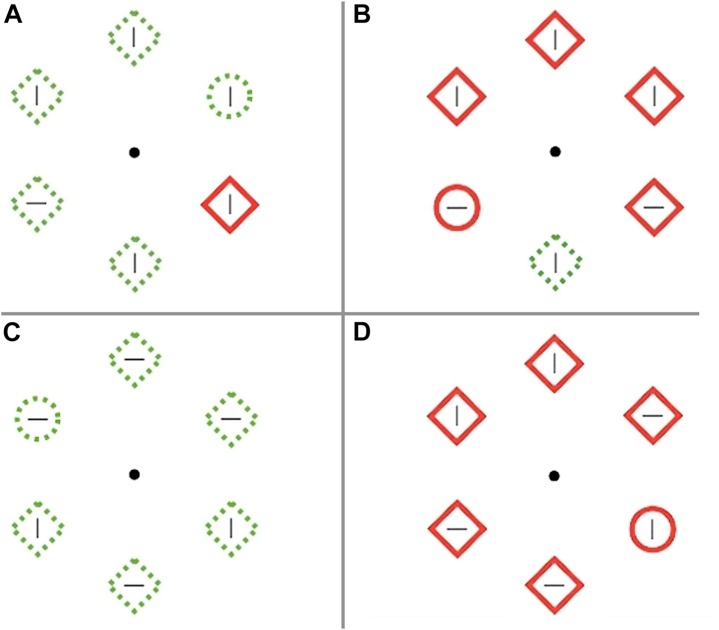
Visual salience task. **(A)** High salient singleton distractor with low salient target and other distractors, **(B)** Low salient singleton distractor with high salient target and other distractors, **(C)** No salient distractor with all green dashed stimuli, **(D)** No salient distractor with all red solid stimuli.

The visual salience task was a visual search adapted from similar tasks used to measure ability to suppress salient distractors (Gaspar and McDonald, 2014; Gaspar et al., 2016). A visual search array contained six unfilled shapes with a line in the center of the shape, presented equidistant from a central fixation point. The goal was to locate the target, a circle among distractor diamonds, and indicate whether the target contained a horizontal or vertical line in the center. Participants were instructed to respond as quickly and accurately as possible. Shape continuity and color were manipulated to make the target and distractors of high (solid red shape) or low (dashed green shape) salience. Each distractor contained a line that was congruent (same orientation) or incongruent (opposite orientation) with the target line. There were 4 trial types: high salient singleton distractor among low salient target and distractors, low salient singleton distractor among high salient target and distractors, no salient distractor with all green dashed stimuli, and no salient distractor with all red solid stimuli (see Figure 2). Participants completed a practice block of 75 trials followed by 5 blocks of the task, with 30 s rest between blocks. Each block contained 10 buffer trials before and after 120 critical trials, for a total of 600 critical trials. Each trial lasted 1000 ms with 500 ms between trials.

Order was pseudo-randomized within blocks, with approximately 1/3 high salient distractors, 1/3 low salient distractors, and 1/3 no salient distractors. Accuracy and reaction time were recorded. Higher accuracy and faster reaction times on salient target trials, and lower accuracy and longer reaction times on salient distractor trials reflect greater sensitivity to salience. It was decided *a priori* to exclude participants with performance below 70% and 9 participants were excluded for this reason. High salience effects were calculated as the difference between the high salient distractor and no salient distractor with green stimuli for mean accuracy and reaction time. Low salience effects were calculated as the difference between the low salient distractor and no salient distractor with red stimuli for mean accuracy and reaction time. For example, the high salience reaction time effect represents how much slower participants responded to the target among green diamonds when a red diamond distractor was present versus not.

#### Latent Inhibition

The LI task was a replication of the within-subjects version used by Granger et al. (2012). Participants were shown a series of letters presented for 1000ms each. Letter X was the target; S and H were cues; D, M, T, and V were distractors. One cue was pre-exposed (PE) and the other cue was non-pre-exposed (NPE), counterbalanced across participants. Participants were instructed to respond when they saw the target (yielding a positive reaction time) or before the target was presented when they were able to predict when it would appear next (yielding a negative reaction time). Participants first completed a pre-exposure phase, during which the PE letter was presented amongst other random letters without cueing the target. During the test phase, the PE and NPE letters cued the target and were displayed amongst random distractor letters. There were 30 target trials: 10 non-cued, 10 following the PE cue, and 10 following the NPE cue. Participants were not informed of the difference between PE and NPE cues or when the task shifted from the pre-exposure phase to the test phase. Response time and accuracy were measured. It was decided *a priori* to exclude participants with 7 or more misses and 14 or more false alarms based on standards in the literature (Evans et al., 2007; Granger et al., 2012) and 7 participants were excluded for low performance. The LI effect was computed as the difference between average PE and NPE reaction times.

#### General Cognition

General cognitive ability was measured in a larger test battery as part of a related study; it was included in the current study *post hoc* as a potential covariate at the suggestion of a reviewer. General cognition was estimated using one crystallized and three fluid intelligence tasks. Crystallized intelligence was assessed using a 42-item form of the Shipley-2 Vocabulary test (Shipley et al., 2009). Fluid intelligence was assessed using the 13-item Cattell Culture Fair-III series subtest (Cattell, 1949), 13-item Ravens Advanced Progressive Matrices (Raven et al., 2004), and 10-item Educational Training Service Paper Folding task (Ekstrom et al., 1976).

### Statistical Method

Pearson correlations were computed to assess predicted associations among salience and LI measures. Positive and negative schizotypy were simultaneously entered in linear regression analyses of salience and LI measures. The positive x negative schizotypy interaction term was entered at a second step (over-and-above the schizotypy main effects) to predict salience and LI measures.

General cognition was estimated by creating latent variables with Mplus8 (Muthén and Muthén, 1998-2017), using maximum likelihood estimation. A fluid intelligence factor was indicated by the three fluid reasoning tasks and the variance was fixed to one. A crystallized intelligence factor was indicated by the vocabulary task and the variance was fixed to one. A second-order general cognitive factor was indicated by the fluid and crystallized intelligence factors and the variance was fixed to one.

## Results

### Descriptive Statistics

Table 1 presents descriptive information for salience measures, LI, general cognition, and schizotypy scales (M, SD, range). The mean positive schizotypy factor score was 0.09 (SD = 1.00), with a range of −1.08 to 3.06. The mean negative schizotypy factor score was 0.41 (SD = 1.03), with a range of −1.07 to 3.70, indicating a broad range of scores on the schizotypy dimensions. Average scores on the brief schizotypy scales were comparable to those reported in two large samples of young adults (Gross et al., 2012). On the illusory contingency task, participants successfully got the cheese on 63% of trials, providing adequate opportunities to develop and test hypotheses about causation. Fourteen participants (16.7%) guessed the correct rule unprompted and 31 participants (36.9%) chose the correct rule from the list of possible rules. Participants’ success rate was unassociated with whether they guessed the correct rule (*r* = 0.20), how many incorrect rules they listed (*r* = 0.01), how many rules they tested and rejected (*r* = −0.02), and how many rules they believed in without testing (*r* = −0.05). Success rate was associated with confidence in the incorrect rules listed (*r* = 0.45, *p* < 0.001), meaning that the more often a participant got the cheese, the more strongly they believed that their interpretation of the rules was correct. General cognition was unassociated with all schizotypy dimensions and outcome measures (*n* = 72: all −0.15 < *r* < 0.12, *p* < 0.22); thus, it was not included as a covariate in any analyses. As a manipulation check, paired samples *t*-tests were run to examine basic effects across participants for LI and visual salience tasks. Cohen’s d effect sizes are reported. Following Cohen (1992), effects sizes of 0.2 are considered small, 0.5 are medium, and 0.8 represent large effects. Comparison of PE and NPE reaction times indicated a significant LI effect overall, *t*(83) = −3.52, *p* < 0.001, *d* = 0.38. On the visual salience task, high salience effects were significant for accuracy, *t*(83) = −7.88, *p* < 0.001, Cohen’s *d* = 0.86, and reaction time, *t*(83) = −2.99, *p* = 0.004, Cohen’s *d* = 0.33. Low salience effects were significant for accuracy, *t*(83) = −4.01, *p* < 0.001, Cohen’s *d* = 0.44, but not reaction time, *t*(83) = 0.12, *p* = 0.90, Cohen’s *d* = 0.01. High salience effects were significantly stronger than low salience effects for accuracy, *t*(83) = −4.01, *p* < 0.001, *d* = 1.20, and reaction time, *t*(83) = −3.11, *p* = 0.003, *d* = 0.34.

**TABLE 1:** Descriptive information on study measures.

	***n***	**Mean (SD)**	**Min**	**Max**
**Wisconsin schizotypy scales—short forms**				
Perceptual aberration	84	1.2 (2.1)	0	10
Magical ideation	84	3.8 (3.2)	0	12
Physical anhedonia	84	3.0 (2.3)	0	9
Social anhedonia	84	2.5 (2.6)	0	12

**General cognition**				
Vocabulary	72	16.8 (4.3)	7	26
Series	72	7.3 (1.9)	2	10
Advanced progressive matrices	69	6.0 (1.5)	3	9
Paper folding	69	4.9 (2.8)	0	10

**Latent inhibition**				
Average non-pre-exposed RT (ms)	84	352.2 (91.9)	50.8	568.0
Average pre-exposed (ms)	84	384.7 (44.8)	238.2	526.0
LI reaction time effect (ms)	84	32.5 (84.5)	−90.8	462.7

**Visual salience**				
High salience accuracy effect	84	−0.05(0.06)	−0.2	0.1
Low salience accuracy effect	84	−0.02(0.05)	−0.2	0.1
High salience RT effect	84	−124.8(52.1)	−12.0	37.4
Low salience RT effect	84	−72.9(63.2)	0.95	26.1

**Illusions of contingency task**				
Number of successful trials	84	31.7 (26.3)	0	88
Average time per trial	84	6.9 (1.1)	3.1	1.4
Number of ineffective key presses	84	44.3 (65.2)	1	476
Average effective path length	84	8.7 (4.1)	4.0	20.3
% participants who guessed correct rule	84	15.5%	–	–
Number of incorrect rules listed	84	1.2 (0.9)	0	4
Confidence in incorrect rules	67	4.8 (1.8)	1	7
Number of rules considered but not tested	84	3.5 (1.8)	0	8
Number of incorrect rules tested, ruled out	84	1.5 (1.4)	0	5

**Aberrant salience inventory**	**84**	15.5(7.0)	**2**	**29**

### Associations Among Measures

Table 2 presents correlations among salience measures. Measures were generally unassociated across levels of processing: negligible to small effects were found among visual, behavioral, and experiential measures of salience. The number of incorrect rules listed on the illusory contingency task was the only variable significantly associated with self-report of aberrant salience experiences. Table 3 presents correlations of LI with salience measures. LI was not associated with salience measures at any level of processing (negligible to small effect sizes).

**TABLE 2:** Correlations among salience measures (*N* = 84).

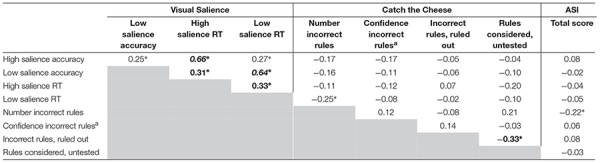

*ASI, aberrant salience inventory. ^∗^*p* < 0.05. Medium effects in bold. Large effects in bold italics. ^*a*^*N* = 67 for participants who listed confidence in incorrect rules following the illusory contingency task.*

**TABLE 3:** Correlations of salience measures with LI (*N* = 84).

	**Correlation with LI effect (*r*)**
High salience accuracy effect	0.10
Low salience accuracy effect	0.03
High salience RT effect	0.08
Low salience RT effect	0.20

Number of incorrect rules	–0.03
Confidence in incorrect rules^a^	0.09
Number of incorrect rules tested and ruled out	–0.004
Number of rules considered but not tested	0.03

Aberrant Salience Inventory	–0.04

*All *p* > 0.05. LI = latent inhibition. ^*a*^*N* = 67 for participants who listed confidence in incorrect rules following the illusory contingency task.*

### Regression Analyses

Multiple regression examined outcomes of salience and LI measures on positive and negative schizotypy simultaneously at step one and the interaction of positive x negative schizotypy at step two. For these analyses, assumptions for multiple regression were mostly met, with the following exceptions: the residuals for the illusory contingency variables and LI were not normally distributed. Residuals were normally distributed in all other cases. Log, natural log, and square root transformations of these variables did not yield normally distributed residuals from regression analyses so non-transformed data was used. Other assumptions of multiple regression were met: for all outcomes, the relationships between independent and dependent variables were linear, the residuals were independent (no auto-correlation), residuals were homoscedastic, and there were no significant outliers. To examine the impact of multicollinearity, variance inflation factor (VIF) was computed for positive schizotypy, negative schizotypy, and the interaction term, following Aiken et al. (2003). All VIF values were less than 1.1, indicating that multicollinearity did not adversely impact the regression analyses.

Table 4 presents regression analyses. For simultaneous regression analyses, as hypothesized, ASI scores were significantly predicted by positive schizotypy (large effect size) and inversely predicted by negative schizotypy (small effect size). Neither positive nor negative schizotypy predicted any visual salience, contingency illusion, or LI variables. The positive x negative schizotypy interaction term did not predict ASI scores, number of incorrect rules listed, or number of rules considered but not tested.

**TABLE 4:** Regressions of positive and negative schizotypy predicting salience variables and latent inhibition (*N* = 84).

	**Step 1 (*df* = 2,81)**						**Step 2 (*df* = 1,80)**			
			
	**Positive schizotypy**			**Negative schizotypy**			**Positive x negative schizotypy**			
				
**Criterion**	**β**	**Δ*R*^2^**	***f*^2^**	**β**	**Δ*R*^2^**	***f*^2^**	**β**	**Δ*R ^2^***	***f*^2^**	***Total R^2^***
High salience accuracy effect	–0.001	0.00	0.00	–0.01	0.00	0.00	–0.18	0.03	0.03	0.03
Low salience accuracy effect	–0.08	0.01	0.01	0.15	0.02	0.02	–0.21	0.04	0.05	0.07
High salience RT effect	–0.09	0.01	0.01	0.08	0.01	0.01	–0.03	0.001	0.001	0.01
Low salience RT effect	–0.07	0.01	0.00	0.01	0.00	0.00	–0.12	0.01	0.01	0.02

Latent inhibition RT effect	–0.08	0.01	0.01	–0.16	0.03	0.03	0.10	0.009	0.009	0.04

Incorrect rules listed	–0.10	0.01	0.00	0.15	0.02	0.01	–0.003	0.00	0.001	0.03
Confidence in incorrect rules^a^	0.06	0.00	0.00	–0.10	0.01	0.00	–0.06	0.003	0.003	0.02
Incorrect rules, ruled out	0.03	0.00	0.01	–0.02	0.00	0.02	0.03	0.001	0.001	0.002
Rules considered, untested	0.01	0.00	0.00	–0.11	0.01	0.01	0.05	0.003	0.003	0.01

Aberrant salience inventory	0.67^∗∗^	0.44	***0.82***	−0.20^∗^	0.04	0.07	–0.08	0.006	0.01	0.47

*^∗^*p* < 0.05, ^∗∗^*p* < 0.001. Large effect sizes in bold italics. RT, reaction time. ^*a*^*N* = 67; Step 1 *df* = 2,64; Step 2 *df* = 1,63 for participants who listed confidence in incorrect rules following the illusory contingency task.*

### *Post hoc* Analyses

Because past research found associations specifically using the Magical Ideation Scale, *post hoc* analyses tested associations of magical ideation with salience variables across levels of processing. Magical ideation showed the same pattern as the overall positive schizotypy factor: it was associated with the ASI at the level of a large effect size (*r* = 0.68, *p* < 0.001) but was not associated with illusions of contingency or visual salience. In comparison, the Perceptual Aberration Scale correlated with the ASI at the level of a medium effect size (*r* = 0.38, *p* < 0.001). *Post hoc* analyses (Supplementary Tables S1–S3) were also conducted including the 14 participants who were excluded for low performance on LI and/or visual salience tasks. The low-performing individuals did not differ in terms of age *t*(96) = 0.90, *p* = 0.39, *d* = 0.32; gender χ*2*(1) = 0.03, *p* = 0.86, Φ = 0.02; positive schizotypy scores *t*(96) = −0.84, *p* = 0.40, *d* = 0.33; or negative schizotypy scores *t*(96) = −0.42, *p* = 0.67, *d* = 0.14; although they differed somewhat by ethnicity χ*2*(5) = 14.76, *p* < 0.05, Φ = 0.40. When including low-performing individuals for a total of 98 participants, results did not change appreciably for correlations or hierarchical linear regression analyses.

## Discussion

The current study examined aberrant salience in positive and negative schizotypy using a variety of methodological approaches across perceptual, behavioral, and cognitive levels of processing. Results replicated findings by Cicero et al. (2010) that trait dimensions show differential associations with ASI scores: self-reported experiences of aberrant salience were positively associated with positive schizotypy and inversely associated with negative schizotypy.

The content of the ASI is similar to certain positive schizotypy scale items, particularly the Magical Ideation Scale. In our sample, ASI scores showed large associations with magical ideation and medium associations with perceptual aberration, comparable to effects in an undergraduate sample from the initial ASI validation study (Cicero et al., 2010). Nonetheless, magical ideation and aberrant salience represent distinct constructs. Compared to the Magical Ideation Scale, the ASI captures experiences that are more emotional in nature, harkening back to the prodromal period of delusional mood described by early phenomenologists such as Conrad (1958, Mishara, 2010). Conrad depicted patients’ impending sense that their environment had somehow fundamentally changed and that something important was about to happen, capturing the tension, excitement, and fear that often accompany the period of transition into psychosis. Kapur (2003) drew on these concepts in his seminal paper on aberrant salience, relating excessive dopamine release that occurs out of context to the feeling of heightened awareness and significance preceding psychotic symptoms, namely, the experience of apophenia described by Conrad (1958). It is important to identify mechanisms underlying salience processing; however, a purely cognitive theory would fall short in failing to account for the marked alterations in mood and affect that accompany experiences of aberrant salience in schizotypy. Kapur’s model moves beyond theories of “cold” cognitive processing by emphasizing the anxiety and emotionality that precede psychotic symptoms. Indeed, experience-sampling studies have shown that momentary negative affect predicts experiences of aberrant salience at the next time point in patients with psychosis (So et al., 2018). Taken altogether, this suggests that cognition and emotion are both important to consider in theoretical models of aberrant salience.

As expected, negative schizotypy was associated with diminished reports of aberrant salience. This is consistent with past psychophysiological and auditory illusion studies that have reported diminished salience in association with negative schizotypy traits (Galdos et al., 2010; Balog et al., 2013). Findings are also consistent with neuroscience theories of deficits in reward processing and motivation in negative symptoms of schizophrenia (Heinz and Schlagenhauf, 2010; Gradin et al., 2013). In this sense, the negative symptom experience of salience is antithetical to that of positive symptoms – the world is experienced as duller and more distant, with diminished associations. As described by Bleuler, 1911, p. 10), “The connections between associations are lost. The disease interrupts the threads that give direction to our thoughts in an irregular fashion, sometimes affecting only a few, sometimes a large proportion of them” (translation, Maatz et al., 2015, p. 45).

Unexpectedly, neither positive nor negative schizotypy showed associations with visual salience or contingency illusions. The effect sizes were miniscule, suggesting that this was not simply an issue of insufficient power in the study. The results were identical with a combined positive schizotypy factor including both perceptual aberration and magical ideation, and with magical ideation alone. This is consistent with other findings in the literature that schizotypy was not associated with greater sensitivity to visual salience on behavioral tasks (Tsakanikos, 2004; Kornmayer et al., 2015). However, our results contrast previous findings that people high in magical ideation or superstitiousness are more likely to believe in spurious causality (Brugger and Graves, 1997; Griffiths et al., 2018). The lack of replication is not likely due to low power given that Brugger and Graves (1997) found a medium-large effect (Cohen’s *d* = 0.72) with 20 people each in high versus low magical ideation groups. Schizotypy scores on the short scales in the current study and magical ideation scores on the full scale in Brugger and Graves’ study were both comparable to those reported in two large samples of young adults, in which schizotypy scores of that level predicted interview measures of psychotic-like and schizotypal symptoms and functional impairment (Gross et al., 2012). Therefore, the level of schizotypy traits in our study was sufficient to examine associations with salience measures.

The current study used self-report measures of schizotypy traits in a university sample and it is unclear whether findings would generalize to community or clinical samples. However, significant salience effects across participants suggested that task manipulations were successful; these outcomes simply did not differ across levels of schizotypy in our sample. Thus, it is possible that salience processing is not disrupted until later on in the course of illness, such as during the prodromal period described by early phenomenologists. Several studies have assessed salience processes in individuals at clinical high-risk (CHR) for psychosis who are experiencing attenuated psychotic symptoms or declined functioning in the context of genetic risk or personality psychopathology. The literature has shown aberrant salience processing in CHR samples using a variety of paradigms, including neurobiological, behavioral, self-report, and experience-sampling studies (Roiser et al., 2013; Modinos et al., 2015; Reininghaus et al., 2016; Pelletier-Baldelli et al., 2018). For example, CHR participants were more likely than control participants to rate irrelevant stimulus properties as associated with reward on a behavioral task and showed diminished associations between functional ventral striatal response and reward predictions during the task (Roiser et al., 2013). Thus, although aberrant salience may still be present before the onset of psychosis, it seems that effects are more robust in clinical manifestations of schizotypy.

In the current study, salience measures were generally unassociated across levels of processing: negligible to small effects were found among visual, cognitive, and self-report measures of salience. Our findings suggest that “aberrant salience” as described broadly in the literature likely involves several dissociable processes (e.g., pre-attentive, perceptual, cognitive, and affective processes), some of which may not show major disruption in subclinical individuals. The links among these constructs are likely to remain murky until mechanisms that give rise to aberrant salience are clearly identified. Current results showed that LI was not associated with salience measures at any level of processing, raising questions about the way in which pre-attentive processes may account for aberrant beliefs and experiences across the schizotypy spectrum. The particular visual salience and LI tasks used in our study did not reveal significant associations with reported aberrant salience experiences. However, the current study was not able to test all possible mechanisms described in predictive coding models (e.g., Fletcher and Frith, 2009) so we cannot rule out that pre-attentive and attentive processes may contribute to or exacerbate aberrant salience. In our study, positive schizotypy traits and generation of incorrect hypotheses were associated with self-report of aberrant salience experiences. Thus, attenuated experiences of aberrant salience in subclinical groups may represent schizotypal personality traits that manifest as a tendency toward magical thinking and reasoning biases that lead to perceived connections among events that are not likely related.

## Conclusion

The aberrant salience hypothesis provides an important contribution to our theoretical understanding of atypical experiences in positive schizotypy and diminished internal experiences in negative schizotypy. However, the broad use of the term aberrant salience is not helpful when applied to describe outcomes across various levels of processing that seem to involve different mechanisms. Instead, clear definitions of the construct and theoretical models being used can make it easier to relate findings across scientific fields. Mechanisms underlying salience processing remain unclear and the current findings did not support a strong role for pre-attentive processes. Inclusion of social and affective factors in addition to cognitive variables is recommended for future study.

## Ethics Statement

This study was approved by the Institutional Review Board (IRB) of the University of North Carolina at Greensboro and carried out in accordance with the recommendations of the IRB. All subjects gave written informed consent in accordance with the Declaration of Helsinki.

## Author Contributions

CC contributed to project conceptualization, project administration, methodology, data curation, formal analysis, interpretation, and manuscript writing, review, and editing. PB contributed to methodology, software, interpretation, and manuscript review and editing. TK contributed to project conceptualization, methodology, formal analysis, resources, supervision, interpretation and manuscript writing, review, and editing.

## Conflict of Interest Statement

The authors declare that the research was conducted in the absence of any commercial or financial relationships that could be construed as a potential conflict of interest.
