# Supplementary material for: Aberrant Salience Across Levels of Processing in Positive and Negative Schizotypy
Source: Front Psychol. 2019 Sep 18;10:2073. doi: 10.3389/fpsyg.2019.02073 (PMC6759779; doi:10.3389/fpsyg.2019.02073)
Supplement: Supplementary file 1 [file Data_Sheet_1.PDF]

Supplemental Materials

Table S1
Correlations among salience measures including participants with low performance (N = 98).

	Visual Salience			Catch the Cheese				ASI
	Low salience accuracy	High salience RT	Low salience RT	Number incorrect rules	Confidence incorrect rules[a]	Incorrect rules, ruled out	Rules considered, untested	Total score
High salience accuracy	.24*	***.68*** ***	.25*	-.18	-.15	-.06	-.04	.08
Low salience accuracy		.29**	***.58*** ***	-.14	-.12	-.09	-.04	-.04
High salience RT			**.34****	-.14	-.07	.03	-.14	-.02
Low salience RT				**-.30****	-.05	-.04	-.10	-.06
Number incorrect rules					.09	-.07	.25*	-.13
Confidence incorrect rules[a]						.21	-.03	.11
Incorrect rules, ruled out							**-.33****	.08
Rules considered, untested								-.02

ASI = Aberrant Salience Inventory. **p* < 0.05. ***p* < 0.01. Medium effects in bold. Large effects in bold italics. [a]N = 80 for participants who listed confidence in incorrect rules following the illusory contingency task.

Table S2

Correlations of salience measures with LI including participants with low performance (N = 98).

	Correlation with LI effect (*r*)
High salience accuracy effect	.05
Low salience accuracy effect	.07
High salience RT effect	.06
Low salience RT effect	.19
Number of incorrect rules	-.11
Confidence in incorrect rules[a]	.03
Number of incorrect rules tested and ruled out	-.04
Number of rules considered but not tested	.02
Aberrant Salience Inventory	-.08

All $p > 0.05$. LI = Latent Inhibition. [a]N = 80 for participants who listed confidence in incorrect rules following the illusory contingency task.

Table S3.

Regressions of positive and negative schizotypy predicting salience variables and LI including participants with low performance (N = 98).

	**Step 1** (df=2,95)						**Step 2** (df=1,94)			
	Positive Schizotypy			Negative Schizotypy			Positive x Negative Schizotypy			
**Criterion**	**β**	ΔR^2	f^2	**β**	ΔR^2	f^2	**β**	ΔR^2	f^2	*Total* R^2
High salience accuracy effect	-.02	.00	.00	.01	.00	.00	-.13	.02	.02	.02
Low salience accuracy effect	-.06	.003	.003	.14	.02	.02	-.20	.04	.04	.06
High salience RT effect	-.05	.002	.002	.09	.007	.007	.00	.00	.00	.009
Low salience RT effect	-.02	.001	.001	.02	.00	.00	-.10	.009	.009	.01
Latent inhibition RT effect	-.12	.02	.02	-.16	.03	.03	.06	.004	.004	.05
Incorrect rules listed	-.01	.00	.00	.08	.007	.007	-.04	.001	.001	.008
Confidence in incorrect rules[a]	.08	.006	.006	-.11	.01	.01	-.11	.01	.01	.03
Incorrect rules, ruled out	.05	.002	.002	-.001	.00	.00	.03	.001	.001	.003
Rules considered, untested	.04	.001	.001	-.12	.02	.02	.06	.004	.004	.02
Aberrant Salience Inventory	.64**	.40	***.72***	-.21*	.04	.08	-.08	.007	.01	.44

*p < 0.05, **p < 0.001. Large effect sizes in bold italics. LI = Latent Inhibition. RT = Reaction Time. [a]N = 80; Step 1 df = 2,77; Step 2 df = 1,76 for participants who listed confidence in incorrect rules
